# Sex-Specific Structural Vulnerability in Alzheimer’s Disease: Insights from APOE ε 4-Negative Patients

**DOI:** 10.3390/life16081290

**Published:** 2026-08-05

**Authors:** Wanessa Michelin, Joana O. Pinto, Bruno Peixoto

**Affiliations:** 1 Department of Social and Behavioral Sciences, University Institute of Health Sciences—CESPU, 4585-116 Gandra, Portugal; a42013@alunos.cespu.pt (W.M.); joana.pinto@iucs.cespu.pt (J.O.P.); 2 Associate Laboratory I4HB, Institute for Health and Bioeconomy, University Institute of Health Sciences—CESPU, 4585-116 Gandra, Portugal; 3 UCIBIO—Applied Molecular Biosciences Unit, Translational Toxicology Research Laboratory, University Institute of Health Sciences—CESPU, 4585-116 Gandra, Portugal; 4 Laboratory of Neuropsychophysiology, Faculty of Psychology and Education Sciences, University of Porto, 4200-135 Porto, Portugal

**Keywords:** genotype, precision medicine, brain atrophy, neurocognitive disorder, Alzheimer’s disease, APOE

## Abstract

**Background:** The interaction between sex, APOE ε4 status, and clinical progression in Alzheimer’s Disease (AD) remains a subject of debate. While females are often considered at higher risk for AD, the underlying structural neuroanatomical trajectories and how they are modulated by genotype are not fully elucidated. This study aims to evaluate how sex and the APOE ε4 genotype interact to influence longitudinal brain atrophy across three clinical groups. **Methods:** We analyzed longitudinal data from 2400 participants from the Alzheimer’s Disease Neuroimaging Initiative (ADNI), stratified by clinical group (i.e., cognitively normal, mild cognitive impairment, and AD), sex, and APOE ε4 carrier status. Using Type III Sum of Squares ANCOVA, we modeled the longitudinal variation in brain volume, controlling for baseline brain volume, and baseline severity of neurocognitive impairment and age at entry. **Results:** While main effects of sex and APOE genotype were not significant, the triple interaction (APOE * Sex * Clinical Group) was marginally significant (*p* = 0.051). Post hoc analysis revealed a distinct pattern of structural dimorphism within the AD cohort among APOE ε4-negative individuals with females exhibiting significantly greater structural preservation compared to males (Mean difference = 11.32, *p* = 0.051). Among APOE ε4 carriers, atrophy trajectories for males and females were statistically indistinguishable (*p* = 0.922), potentially suggesting that the ε4 allele exerts a dominant neurodegenerative influence that overrides sex-specific physiological differences. **Conclusions:** These emerging findings highlight the importance of jointly considering biological sex and APOE ε4 status to improve the characterization of Alzheimer’s disease heterogeneity and support precision medicine approaches.

## 1. Introduction

Alzheimer’s disease (AD) is the leading cause of dementia worldwide, accounting for 60 to 70% of all cases and presenting a major global public health challenge [1]. Among the factors contributing to AD vulnerability, epidemiological, neurobiological, and genetic factors interact, with sex representing a key determinant across these domains.

Epidemiologically, AD displays a marked asymmetry, with women representing nearly two-thirds of those living with the disease [2], and showing a higher lifetime risk of developing AD, estimated at approximately 21% at age 65 compared to 12% in men [2]. While this disparity was historically attributed to women’s longer life expectancy, recent evidence indicates that it also reflects distinct, sex-specific biological mechanisms [3]. This recent perspective aligns with the concept of “dimorphic neurology” proposed by Rocca et al. [4], which argues that biological sex influences the risk, etiology, and progression of major neurocognitive disorder through divergent mechanisms, including hormonal profiles and different brain aging trajectories.

Neurobiologically, differences have been reported in patterns of brain atrophy, particularly in regions such as the hippocampus and medial temporal lobe, as well as in susceptibility to tau pathology [3,5]. In addition, neuroimaging studies have demonstrated that women with amnestic mild cognitive impairment (MCI) and AD exhibit faster rates of brain atrophy than men, with annual rates exceeding those of men by 1 to 1.5% per year [6]. Multiple factors have been proposed as drivers of these disparities. These include hormonal changes, such as the decline in estrogen levels associated with menopause, as well as differences in neuroinflammatory responses, synaptic resilience, and metabolic regulation [3]. Therefore, recognizing biological sex as a driver of disease risk and progression, rather than merely a demographic covariate, is essential for improving diagnostic accuracy and tailoring Alzheimer’s assessments [7].

Genetic factors also contribute significantly to AD vulnerability. Among them, the apolipoprotein E (APOE) gene represents the strongest known genetic risk factor for late-onset sporadic AD [8,9]. APOE plays a central role in cerebral lipid transport and neuronal maintenance, contributing to cholesterol homeostasis, synaptic plasticity, neuronal repair, and the regulation of amyloid-β metabolism [8,9]. Three major APOE isoforms have been identified in humans (ε2, ε3, and ε4), among which the ε3 allele is the most common. However, the ε4 allele is overrepresented among AD patients, with approximately 60 to 80% of affected individuals carrying at least one ε4 allele [9]. This risk follows a dose-dependent effect. Heterozygous carriers (ε3/ε4) have a 2–3-fold increased risk, while homozygous carriers (ε4/ε4) show a 10–15-fold increased risk and earlier disease onset. In contrast, the ε2 allele is generally protective. It is associated with reduced amyloid and tau pathology and slower neurocognitive decline in aging populations [10].

APOE ε4 influences several pathological processes. At the molecular level, APOE ε4 impairs Aβ clearance across the blood–brain barrier, and is associated with increased tau pathology, neuroinflammation, mitochondrial dysfunction, and synaptic impairment [8,9]. These alterations lead to widespread neurodegeneration. At the structural level, studies show greater medial temporal lobe atrophy in ε4 carriers, particularly in the hippocampus and parahippocampal regions, following a dose-dependent pattern [11,12]. Additional alterations include entorhinal cortex thinning and later involvement of posterior cingulate and subcortical regions, alongside microstructural white matter changes [13]. At a functional level, these structural alterations are associated with accelerated neurocognitive decline, initially affecting episodic memory and later extending to executive and language functions as pathology spreads [10,14]. Overall, APOE influences AD pathology across molecular, structural, and functional levels. However, interindividual variability in the effects of APOE ε4, suggests that additional factors can modify the individual’s trajectory.

Among the factors that may contribute to this interindividual variability, biological sex has emerged as one of the most consistently reported sources of variability. Female APOE ε4 carriers face a disproportionately higher risk of developing AD compared to male carriers, an asymmetry that cannot be explained by differences in longevity alone [15,16]. The association between APOE genotype and AD risk appears to be significantly influenced by both age and sex, with the effects of the ε4 allele being particularly pronounced in women between 65 and 75 years of age [16].

Sex also appears to influence brain changes, though not across all of the disease stages. Female ε4 carriers may exhibit more pronounced cortical thinning and accelerated atrophy in regions vulnerable to AD pathology, including temporal, frontal, parietal, and limbic areas [17]. Moreover, microstructural alterations associated with APOE ε4, particularly within the entorhinal cortex, have been observed in women but not consistently in men among cognitively normal older adults, pointing to a potential female-specific vulnerability in one of the earliest regions affected by AD [13]. This stage-dependence is particularly evident for hippocampal volume, with ε4 carriage being associated with smaller hippocampal volume in cognitively normal men only, whereas in individuals with MCI the same association emerged across both sexes [18]. This indicates that the sex-by-genotype effect on brain structure shifts as the disease progresses rather than remaining fixed [19].

The influence of sex extends beyond molecular and structural alterations to the functional level, affecting the relationship between AD pathology and neurocognitive performance. At the cognitive level, APOE ε4 carriers face a disproportionately higher risk of neurocognitive impairment compared to non-carriers, with a significantly greater impact in females than males [20]. However, evidence regarding the effect of sex on neurocognitive decline remains mixed. For instance, one study found that sex did not directly influence the rate of neurocognitive decline as a main effect. Nevertheless, females with higher amyloid burden exhibited faster decline than males, and this sex-specific vulnerability was most pronounced among women who were both amyloid-positive and ε4 carriers. This suggests that the relationship between sex, genotype, and neurocognitive outcomes operates through interactions with pathological burden rather than as an independent effect [21]. While neurocognitive performance often appears preserved in women in the early stages, decline may accelerate rapidly once neurodegeneration crosses a critical threshold [5,21]. This discrepancy between brain pathology and neurocognitive function challenges standard neuropsychological assessment, as women often maintain higher verbal memory scores than men despite equivalent pathology, potentially leading to an underestimation of impairment in women and an overestimation in men [19]. Consistent with sex-specific trajectories of neurocognitive aging [22,23], these findings suggest that similar neurocognitive performance may not reflect equivalent underlying disease progression in women and men [24], with important implications for the diagnostic and prognostic value of neuropsychological measures alone.

Taken together, these preliminary findings establish sex as a critical modifier of APOE-related vulnerability in AD, influencing both the severity and distribution of neurodegeneration. Structural neuroimaging findings and neurocognitive outcomes do not always converge, and reported sex-by-genotype interactions vary considerably across disease stages and cohorts [2,15]. More broadly, the risk associated with APOE genotype itself varies significantly with both age and sex across adulthood [25]. This heterogeneity is combined by persistent methodological limitations. Although women constitute the majority of individuals affected by AD, sex-stratified analyses remain underrepresented in both observational studies and clinical trials, and studies continue to treat biological sex as a statistical covariate rather than as a biologically meaningful variable capable of modifying disease trajectories [7]. In response to these gaps, Nebel et al. [26] explicitly identified the integration of sex and gender into neuroimaging and neurocognitive assessment as a research priority. Although recent studies have explored the effects of sex and APOE ε4 on regional patterns of atrophy using complex computational modeling [16], there remains a gap in our understanding of how these effects translate into global volumetric variations, which are metrics that are more readily applicable to clinical practice and routine diagnosis.

The primary objective of this study is to evaluate how biological sex and the APOE ε4 genotype interact to influence longitudinal brain atrophy across three clinical groups: cognitively normal (CN) individuals, patients with MCI, and those with AD. Secondarily, this study aims to assess whether the interaction between sex and APOE status manifests differentially across these clinical diagnostic groups.

## 2. Method

### 2.1. Participants

Data were obtained from the Alzheimer’s Disease Neuroimaging Initiative (ADNI) database (adni.loni.usc.edu). Detailed information regarding the eligibility criteria considered has been published previously [27]. Participants were included in this study if they met the following criteria: (a) availability of baseline demographic and clinical information, including age, sex, Mini-mental State Examination score (MMSCORE_baseline), and diagnostic classification; (b) availability of confirmed APOE ε4 genotype data; (c) availability of longitudinal structural magnetic resonance imaging (MRI) data, defined by the presence of a baseline scan and at least one follow-up scan, to enable the calculation of the longitudinal whole brain atrophy rate (BRAINVOL_delta).

The final analytic sample comprised 2400 individuals, representing a harmonized dataset across ADNI study phases (ADNI-1, ADNI-2, ADNI-3/GO). Participants were categorized into three clinical groups at entry: CN; MCI, encompassing early and late MCI cohorts; and AD. Additionally, participants were stratified by sex (male, female) and APOE ε4 carrier status (non-carrier vs. carrier).

Given the longitudinal design of the ADNI phases, the interval between the baseline and the final follow-up assessment varied according to the expected attrition and progression rates of each clinical cohort. The mean follow-up duration was 63.36 months (SD = 55.02) for the CN group, 49.44 months (SD = 48.76) for the MCI group, and 22.73 months (SD = 19.11) for the AD group.

### 2.2. Measures

Clinical diagnosis was established using standardized ADNI neuropsychological protocols. The primary outcome variable was the longitudinal variation in brain volume (BRAINVOL_delta), calculated as the difference between follow-up and baseline MRI measurements, following standardized ADNI image processing protocols (https://adni.loni.usc.edu/data-samples/adni-data/neuroimaging/mri/) (accessed on 2 July 2026), baseline and follow-up structural MRI scans underwent automated segmentation to quantify global volumetric changes. This metric was calculated as the net difference between follow-up and baseline measurements. In practical terms, higher values on this metric reflect greater neuroanatomical preservation (i.e., an attenuated rate of atrophy), whereas lower values indicate more severe structural volume loss over the observational period. APOE genotype was dichotomized based on the presence of at least one ε4 allele, to ensure adequate statistical power and stable subgroup estimation within the multi-way interaction models. Age at entry, baseline brain volume, and baseline MMSE scores (MMSCORE_baseline) were included as covariates to control for the physiological effects of aging, baseline structural reserve, and global neurocognitive impairment severity.

### 2.3. Procedures

The ADNI protocol received ethical approval from the institutional review board at each participating centre, and all participants or their legally authorised representatives provided written informed consent before enrolment. The data used in this study were obtained from the ADNI database on 26 April 2026.

### 2.4. Statistical Analysis

Statistical analyses were conducted using R Studio (version 4.2.5). To assess the impact of sex, APOE status, and clinical group on structural atrophy, we employed an Analysis of Covariance (ANCOVA) using Type III Sum of Squares to account for unbalanced sample sizes. The model was defined as: BRAINVOL_delta ~ APOE_Risk * Sex * Clinical_group + Age + BRAINVOL_baseline + MMSCORE_baseline + Education. Education (years of schooling) was retained a priori in the model regardless of its statistical significance, as it is a widely recognized theoretical proxy for cognitive reserve. Controlling for this variable ensures that the observed structural preservation trajectories are not confounded by baseline differences in socio-educational background and reserve-related mechanisms.

To ensure the mathematical stability of the interaction terms, we evaluated structural multicollinearity using adjusted Variance Inflation Factors (VIFs); all values were well within acceptable limits (VIF < 10). Post hoc pairwise comparisons were conducted using estimated marginal means (emmeans). All statistical analyses were performed with a significance threshold of *α* = 0.05.

## 3. Results

The sample distribution and genotype prevalence (Table 1) confirm the expected increase in APOE ε4 carrier frequency in the AD group.

VIF analysis (Table 2) confirmed that the structural collinearity arising from the triple interaction remained within acceptable limits with values inferior to 5 [28], validating the stability of the regression coefficients.

The longitudinal variation in brain volume was analysed using a Type III Sum of Squares ANCOVA, adjusting for age, baseline volume, and baseline neurocognitive impairment severity (MMSE). Entry age, baseline brain volume, and baseline MMSE were all significant predictors of the atrophy rate (*p* < 0.05), confirming the model’s validity. While main effects of sex (*p* = 0.668) and APOE genotype (*p* = 0.583) were not statistically significant in the overall cohort, the triple interaction (APOE_Risk × Sex × Clinical_group) approached significance (*F*(2, 2290) = 2.79, *p* = 0.051) (Table 3).

**Figure 1:**
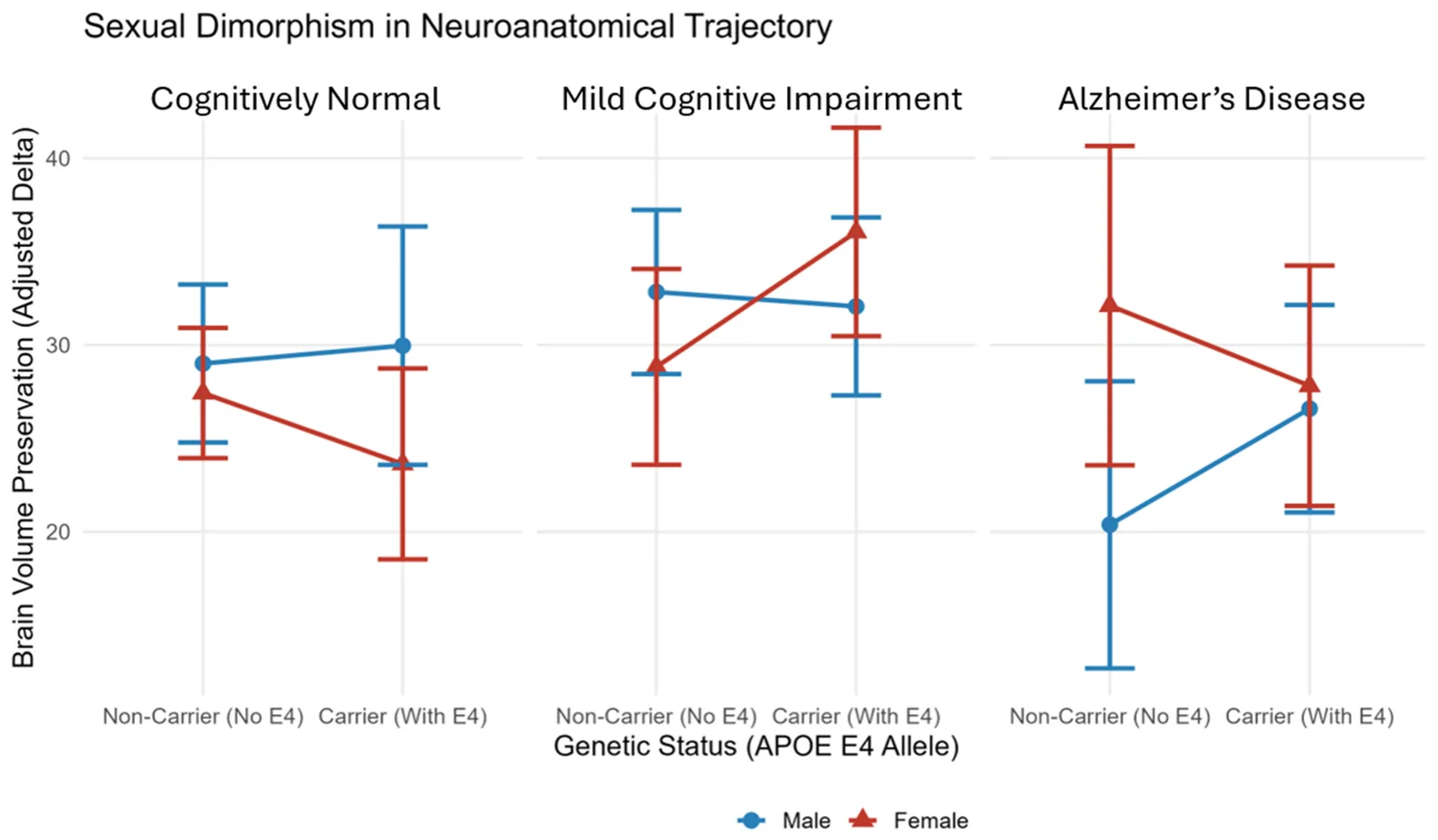
**Sexual dimorphism in neuroanatomical trajectory.** Adjusted marginal means of longitudinal brain volume preservation across clinical groups, stratified by sex and APOE ε4 status. Error bars represent 95% confidence intervals of the adjusted marginal means. Exact sample sizes (n) for each subgroup are detailed in Table 1.

Post hoc analysis revealed a distinct pattern of structural dimorphism within the AD cohort. Among AD patients carrying the APOE ε4 allele, atrophy trajectories were statistically indistinguishable between males and females (*p* = 0.922). Conversely, in the absence of the ε4 allele, a significant divergence emerged. Female participants demonstrated greater volume preservation (Adjusted Mean = 42.6) compared to males (Adjusted Mean = 31.2; Estimate = −11.32, *p* = 0.051; Cohen’s *d* of −0.31 [95% CI: −0.63, 0.01]). This effect persists after controlling for baseline cognitive status (Table 4; Figure 1).

## 4. Discussion

The present study demonstrates a complex interaction, albeit marginally significant, between APOE ε4 status, biological sex, and clinical diagnosis regarding longitudinal brain volume atrophy. While the global interaction warrants cautious interpretation, post hoc analyses revealed that the primary finding is a structural dimorphism observed specifically among non-carriers of the APOE ε4 allele, where females exhibited significantly greater structural preservation compared to males. Conversely, this sexual dimorphism was absent in APOE ε4 carriers, where atrophy trajectories were statistically indistinguishable between sexes. Crucially, these findings persisted after adjusting for baseline global neurocognitive impairment severity (MMSE) and for brain volume at baseline, which is a proxy for cerebral reserve, suggesting that this structural dimorphism is a neuroanatomical phenomenon independent of baseline neurocognitive status.

Consistent with previous evidence, APOE-ε4 appears to modulate the rate of brain atrophy in a sex-specific manner [16]. Whereas previous work focused on regional atrophy patterns, the present study demonstrated that these sex specific effects were also evident in longitudinal global brain atrophy, independent of baseline neurocognitive status and structural reserve. The greater volume preservation observed in female non-carriers aligns with the concept of “dimorphic neurology” [4], suggesting that, in the absence of the ε4 allele, the female brain may possess more robust mechanisms to preserve neuroanatomical integrity against the AD-related pathology. This interpretation is further supported by evidence showing that the effects of APOE on brain microstructure are significantly modified by sex and neurocognitive status, reinforcing the importance of considering sex as a fundamental biological variable rather than a merely nuisance covariate [13]. The disparity in atrophy rates between male and female non-carriers may also be linked to vascular risk factors. Emerging evidence suggests that vascular risk interacts differently with the APOE genotype and sex regarding neurocognitive decline [29]. Accordingly, it is plausible that males are more susceptible to vascular-driven neurodegeneration because they lack the protective or modulatory mechanisms that may exist in females. As baseline neurocognitive status was included as a covariate, this greater vulnerability is unlike to reflect differences in disease severity at study entry, supporting the hypothesis that it may instead represent underlying structural, potentially vascular and hormonal mechanisms [21,22]. However, it is important to explicitly acknowledge that these mechanistic interpretations remain exploratory. While they offer a plausible biological framework for the observed structural dimorphism, these hypotheses warrant further confirmation in future longitudinal studies incorporating specific vascular and endocrine biomarkers. The convergence of atrophy trajectories in male and female APOE ε4 carriers suggests that the ε4 allele acts as a dominant neurodegenerative driver. This genotype may overwhelm the physiological sex-specific protective mechanisms typically observed in structural aging. APOE ε4 status significantly modifies neurocognitive phenotypes, often masking the subtle, sex-specific variations in neurodegeneration [19]. In this context, the ε4 allele functions as a biological “equalizer,” forcing a uniform, accelerated rate of atrophy that renders the intrinsic structural differences between males and females negligible.

These findings underscore the necessity of “disentangling” the effects of sex in Alzheimer’s disease research [19]. Rather than being treated merely as a confounding variable, biological sex should be considered a fundamental determinant of disease heterogeneity, interacting with genetic, hormonal, vascular, and molecular factors to shape neurodegenerative trajectories [6]. This perspective is central to integrative precision medicine, which seeks to characterize the biological heterogeneity of AD by integrating multiple layers of information, including genetic, molecular, imaging, environmental, and lifestyle factors, rather than relying on isolated risk markers [29]. Within this framework, our finding that the APOE ε4 genotype attenuates the structural advantage observed in female non-carriers suggests that sex and genotype should not be examined independently, but as interacting biological determinants of neurodegeneration. These findings therefore support incorporating both sex into multimodal models of AD, potentially improving the biological characterization of disease trajectories and informing more individualized approaches to risk assessment and clinical management.

The main limitation of the present study is that, although it integrated genetic, structural neuroimaging, and clinical information, it did not fully capture the multidimensional nature of AD. Consequently, the clinical significance of the greater structural preservation observed in female non-carriers, its relationship with neurocognitive, neuropsychiatric, and functional manifestations, and the biological mechanisms underlying this finding, including hormonal and vascular factors, remain to be elucidated. Another limitation of the present study is the absence of cerebrospinal fluid (CSF) Aβ and tau biomarker stratification across the full analytic sample, which precludes the biological staging of participants according to the amyloid/tau/neurodegeneration (ATN) framework. However, the ADNI protocol employs highly rigorous and standardized clinical and neuropsychological criteria to establish diagnoses and rule out non-AD causes of dementia [27]. Consequently, while biological confirmation was not included in this model, the pronounced structural atrophy and longitudinal trajectories observed within the AD cohort are consistent with neurodegenerative pathology rather than normative age-related changes. Future investigations incorporating biomarker-stratified, Aβ-positive pure AD cohorts will be valuable to further validate these sex-by-genotype interaction effects. Finally, because our primary outcome metric focuses on global brain volume changes, these conclusions are strictly applicable to overall neurodegeneration and cannot be directly generalized to regional injury patterns in specific structures such as the hippocampus or entorhinal cortex.

Elucidating the mechanisms underlying the greater structural preservation observed in female non-carriers will require future longitudinal studies integrating complementary biological and clinical dimensions of AD. The Multidimensional Model of Neurocognitive Disorders (MOND model) provides one possible conceptual framework for this multidimensional approach by considering the interaction between biological, neurocognitive, neuropsychiatric, functional, reserve-related, and socio-environmental domains [30]. This perspective is aligned with the principles of precision medicine, facilitating the translation of biological discoveries into clinically meaningful characterization of disease heterogeneity. Within this translational framework, neuropsychological assessment represents a key interface between underlying neuropathology and its neurocognitive and functional expression. Accordingly, routinely considering both sex and gender during neuropsychological assessment may further refine clinical characterization, recognizing that sex reflects biological determinants whereas gender encompasses environmental, social, and cultural influences that shape cognitive reserve, clinical presentation, and disease recognition [31].

## Figures and Tables

**Table 1:** Descriptive statistics by clinical group, APOE status, and sex.

Clinical Group	APOE Status	Sex	*n*	Age (*M* ± *SD*)	Education (*M* ± *SD*)	MMSE (*M* ± *SD*)	Brain Volume Baseline (*M* ± *SD*)	Brain Volume Delta (*M* ± *SD*)
CN	Non Carrier	Male	302	73.0 ± 6.5	17.2 ± 2.5	29.4 ± 1.1	1137.9 ± 95.8	33.4 ± 32.6
		Female	420	70.5 ± 7.4	16.1 ± 2.6	29.5 ± 0.8	1021.5 ± 93.4	25.0 ± 30.4
	Carrier	Male	123	71.8 ± 7.1	16.7 ± 2.4	29.4 ± 0.9	1131.1 ± 88.7	33.6 ± 39.2
		Female	191	69.3 ± 6.4	16.3 ± 2.4	29.5 ± 0.9	1031.1 ± 82.6	21.3 ± 23.4
MCI	Non Carrier	Male	266	74.3 ± 7.6	16.1 ± 2.9	28.4 ± 1.7	1110.1 ± 100.6	36.2 ± 62.6
		Female	182	72.3 ± 8.3	15.8 ± 2.6	28.4 ± 1.8	997.4 ± 91.2	25.8 ± 28.6
	Carrier	Male	221	73.8 ± 6.7	16.2 ± 2.8	27.9 ± 2.0	1103.4 ± 103.2	34.9 ± 35.2
		Female	163	71.0 ± 6.9	15.5 ± 2.7	27.8 ± 2.0	989.6 ± 96.2	32.1 ± 35.8
AD	Non Carrier	Male	83	76.3 ± 8.0	15.9 ± 2.8	23.9 ± 2.3	1055.2 ± 94.7	21.7 ± 22.7
		Female	69	76.7 ± 9.3	14.6 ± 2.7	23.8 ± 2.6	945.5 ± 94.5	27.9 ± 32.4
	**Carrier**	Male	159	75.1 ± 7.4	15.9 ± 3.0	23.8 ± 2.4	1069.0 ± 102.6	28.2 ± 26.1
		Female	126	72.4 ± 7.0	14.4 ± 2.7	23.8 ± 2.7	949.0 ± 86.4	22.4 ± 18.1

*Note.* MMSE = Mini-Mental State Examination Data represent mean ± standard deviation (SD). *n* = sample size. Brain volume units are in arbitrary units/cm^3^ (as provided in the ADNI dataset).

**Table 2:** Collinearity diagnosis (Adjusted VIF) for the structural multivariate model.

Variable/Interaction	*df*	Adjusted VIF
APOE_Risk	1	1.06
Sex	1	1.02
Clinical_group	2	1.10
Age	1	1.04
Education	1	1.04
BRAINVOL_baseline	1	1.07
MMSCORE_baseline	1	1.10
APOE_Risk × Sex	1	1.02
APOE_Risk × Clinical_group	2	1.04
Sex × Clinical_group	2	1.01
APOE_Risk × Sex × Clinical_group	2	1.03

*Note.* BRAINVOL = brain volume; MMSCORE = Mini Mental State Examination. VIF = Variance Inflation Factor. Adjusted VIF calculated as GVIF^(1/(2 × *df*))^.

**Table 3:** Analysis of covariance (ANCOVA—Type III) for brain volume preservation.

Source	Sum of Sq	*df*	*F*	*p*
(Intercept)	17.46	1	13.88	**<0.001**
Age	13.01	1	10.35	**0.001**
Education	2927.00	1	39.40	0.124
Baseline Volume	48.06	1	38.23	**<0.001**
MMSCORE_*baseline*	8.24	1	6.55	**0.010**
*Clinical Group*	13.24	2	5.27	**0.005**
APOE Risk	380.00	1	0.30	0.583
*Sex*	231.00	1	0.18	0.668
APOE × Sex × *Clinical_group*	7.01	2	2.79	**0.051**

*Note.* MMSCORE = Mini Mental State Examination. Significant values are shown in bold.

**Table 4:** Post hoc contrast analysis in the clinical three groups.

Group	APOE Status	Mean (Male)	Mean (Female)	Diff (M-F)	*SE*	*p*
CN	Non-Carrier	25.82	22.66	3.16	2.88	0.278
	Carrier	26.50	19.01	7.49	4.18	0.073
MCI	Non-Carrier	31.93	27.09	4.84	3.53	0.170
	Carrier	32.64	35.77	−3.13	3.79	0.408
AD	Non-Carrier	31.2	42.6	−11.32	5.80	**0.051**
	Carrier	37.9	38.4	−0.42	4.34	0.922

Significant values are shown in bold.

## Data Availability

The data analyzed in this study were obtained from the Alzheimer’s Disease Neuroimaging Initiative (ADNI) database (adni.loni.usc.edu). Restrictions apply to the availability of these data, which were used under license for this study. Data are available through application to the ADNI data repository for qualified researchers who meet the criteria for access to confidential data.
